# Association of SARS-CoV-2 genomic load trends with clinical status in COVID-19: A retrospective analysis from an academic hospital center in New York City

**DOI:** 10.1371/journal.pone.0242399

**Published:** 2020-11-17

**Authors:** Ioannis M. Zacharioudakis, Fainareti N. Zervou, Prithiv J. Prasad, Yongzhao Shao, Atreyee Basu, Kenneth Inglima, Scott A. Weisenberg, Maria E. Aguero-Rosenfeld

**Affiliations:** 1 Division of Infectious Diseases and Immunology, Department of Medicine, NYU Grossman School of Medicine, New York, NY, United States of America; 2 Department of Population Health, NYU Grossman School of Medicine, New York, NY, United States of America; 3 Department of Pathology, NYU Grossman School of Medicine, New York, NY, United States of America; University Magna Graecia of Catanzaro, ITALY

## Abstract

The Infectious Diseases Society of America has identified the use of SARS-CoV-2 genomic load for prognostication purposes as a key research question. We designed a retrospective cohort study that included adult patients with COVID-19 pneumonia who had at least 2 positive nasopharyngeal tests at least 24 hours apart to study the correlation between the change in the genomic load of SARS-CoV-2, as reflected by the Cycle threshold (Ct) value of the RT-PCR, with change in clinical status. The Sequential Organ Failure Assessment (SOFA) score was used as a surrogate for patients’ clinical status. Among 457 patients with COVID-19 pneumonia between 3/31/2020-4/10/2020, we identified 42 patients who met the inclusion criteria. The median initial SOFA score was 2 (IQR 2–3). 20 out of 42 patients had a lower SOFA score on their subsequent tests. We identified a statistically significant inverse correlation between the change in SOFA score and change in the Ct value with a decrease in SOFA score by 0.05 (SE 0.02; p<0.05) for an increase in Ct values by 1. This correlation was independent of the duration of symptoms. Our findings suggest that an increasing Ct value in sequential tests may be of prognostic value for patients diagnosed with COVID-19 pneumonia.

## Introduction

The World Health Organization declared Coronavirus Disease 2019 (COVID-19), the disease caused by the severe acute respiratory syndrome coronavirus 2 (SARS-CoV-2), a pandemic on March 11th, 2020 [1]. At the time of this writing, SARS-CoV-2 has infected more than 40 million people and has caused more than 1 million deaths worldwide [2]. Disease severity ranges from asymptomatic or minimally symptomatic infection to hypoxic respiratory failure and death [3]. Early studies have focused on host risk factors to predict adverse clinical outcomes, and elderly patients with comorbidities are considered to have a worse prognosis [4, 5]. Viral factors might also predict disease outcomes, but few clinical studies have focused on elucidating their role. The Infectious Diseases Society of America has recently emphasized that the ability of a quantitative test for SARS-CoV-2 to be used as a prognostic marker remains a key research question [6].

In a previous investigation, we focused on the ability of the SARS-CoV-2 genomic load at the time of admission to the hospital of patients with COVID-19 pneumonia to predict clinical outcomes [7]. We used the readily available Cycle threshold (Ct) value of positive nasopharyngeal samples, i.e. the number of amplification cycles needed to yield a positive fluorescent signal in a real-time reverse transcription-polymerase chain reaction test (RT-PCR), as a surrogate for genomic load. Our results suggested that the genomic load on admission is an independent predictor of a composite outcome of intubation and death. In that study patients were not followed during the disease-course and the possible correlation of genomic load changes with measurable effect in individual’s clinical status was not examined.

In the present study, we aim to examine the association of SARS-CoV-2 genomic load in sequential nasopharyngeal samples with the clinical status of patients on the days of testing. We hypothesized that an increase in Ct values during a patient’s clinical course would be associated with an improvement in their clinical status and vice-versa.

## Methods

### Study design, setting and participants

We conducted a retrospective cohort study at the NYU Langone Medical Center, a tertiary academic medical center in New York City. On March 31^st^, our microbiology laboratory adopted the Cepheid Xpert^®^ Xpress SARS-CoV-2 assay for in-house diagnosis of COVID-19. We evaluated a convenience sample of patients who presented to the emergency department between March 31^st^ and April 10^th^ of 2020 and had 2 or more positive tests for SARS-CoV-2 at least 24 hours apart using this assay. Patients who had a positive repeat test using a different PCR assay were excluded from the final analysis, as targets between different assays differ. The amplification efficiency and dynamic range may also vary, making Ct values derived from different assays not comparable. Twenty-four hours were considered the minimum amount of time where one could observe a meaningful clinical difference. Patients were included in the analysis if they demonstrated clinical (subjective or documented fever, shortness of breath or hypoxia and new or worsening cough) and radiographic findings of pneumonia (airspace or interstitial opacities) during their initial presentation.

### Outcome measures and follow-up

The primary study outcome was the association of the Ct values among patients with repeat testing with their respective Sequential Organ Failure Assessment (SOFA) score, calculated on the day of the respective positive PCR test. We used the SOFA score as it has been shown to provide greater prognostic accuracy for in-hospital mortality than SIRS criteria or quick SOFA score among adults with suspected infection [8]. We followed all patients for 30 days after their initial positive test.

### Real-Time reverse transcriptase Polymerase Chain Reaction (RT-PCR) assay

The Cepheid Xpert^®^ Xpress SARS-CoV-2 assay is a rapid RT-PCR test that has received Emergency Use Authorization by the Food and Drug Administration (FDA) for the qualitative detection of ribonucleic acid (RNA) from SARS-CoV-2 in individuals with suspected COVID-19 [9]. The specimens are processed on the GeneXpert^®^ platform, which automates and integrates sample preparation, nucleic acid extraction and amplification, and detection of the target sequences. The assay detects the nucleocapsid 2 (N2) and Envelope (E) nucleic acids of SARS-CoV-2, and reports their respective Ct values. We used the Ct values for the N2 nucleic acid as this is more specific for SARS-CoV-2 as compared to the E nucleic acid that can also be found in SARS-CoV-1. The lowest limit of detection for this assay is 250 copies/mL [10]. A difference of Ct values of 3.0 represents an approximate order of magnitude difference in genomic load.

### Data collection

We reviewed electronic medical records of included patients and extracted information on demographic characteristics, Body Mass Index (BMI), defined as the patient’s weight in kilograms divided by the square of height in meters, smoking history and respiratory comorbidities including obstructive sleep apnea (OSA), chronic obstructive pulmonary disease (COPD), and asthma. We gathered history of immunocompromising conditions, such as solid organ transplant (SOT) receipt or use of immunosuppressive medications. We used the Charlson Comorbidity Index (CCI) to quantify comorbid conditions and calculated the SOFA scores on the days of initial and repeat positive PCR tests. It has been shown that a minimum difference of 2 points in SOFA scores during hospitalization results in a difference in expected hospital mortality [8]. We extracted the duration of symptoms at the time of initial testing, duration between the repeat PCR tests, and reasons for repeat PCR testing. Patient outcomes, including death or discharge to hospice care, use of mechanical ventilation, discharge home, and readmission were also obtained.

### Statistical analysis

A linear mixed-effects regression analysis was performed to account for within-patient variation and adjust for the number of repeat tests performed per patient. The model was fitted with the restricted maximum likelihood method given the small sample size [11]. In our previous investigation, patients’ Charlson Comorbidity Index, transplant status and duration of symptoms were shown to be significantly associated with Ct values. Given that the mixed-effects model accounts for within-patient variation, and as such for the variation of Ct cycles attributed to comorbidities, duration of symptoms was added in the model to account for the effect of time on the genomic load. All calculations were performed using the Stata v14.2 software package (Stata Corporation, College Station, TX). Results are presented as proportions with 95% confidence intervals (CIs), and medians with interquartile ranges (IQR). A *P* value of <0.05 is considered statistically significant. This study was approved with a waiver of informed consent by the New York University Institutional Review Board.

## Results

**Fig 1:**
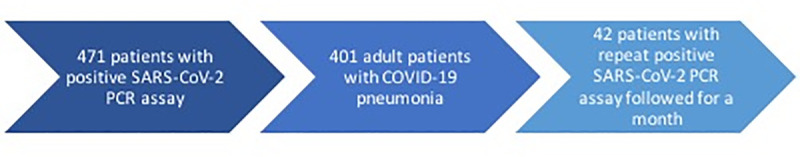
Flow chart.

471 of the patients who presented to our emergency department from March 31^st^ to April 10^th^, 2020, tested positive for SARS-CoV-2. Ten patients who were < 18 years old and 60 who did not exhibit any clinical or radiographic signs of pneumonia were not considered for further analysis. Of the remaining patients, 42 had a repeat positive test with the same assay, at least 24 hours after the original positive screening and within the 1-month follow-up period, and were included in the analysis. The Flow Chart is presented in Fig 1.

Among the 42 included patients, the median age was 67 years (IQR 58–81.75), 29 (69.0%) were male, and had a median BMI of 25.4 (IQR 22.0–30.2). In terms of comorbidities, median Charlson comorbidity index was 4 (IQR 2–5), 10 patients (23.8%) had at least 1 pulmonary comorbidity, with the most common being asthma (6 patients, 14.3%), 13 patients (31.0%) were active or previous smokers, and 5 patients (11.9%) were solid organ transplant recipients (2 kidney, 1 liver, 1 lung, 1 heart/kidney). Table 1 presents the patients’ baseline characteristics.

**Table 1:** Patient characteristics.

**Demographics/Patient Characteristics**	**No. of patients (%) or Median (IQR)**
**Age**	67 (58–81.75)
**Gender**	
Female	13 (31.0%)
Male	29 (69/0%)
**Race**	
White	19 (45.2%)
Afr. American/Black	3 (7.1%)
Asian	6 (14.3%)
Hispanic	2 (4.8%)
Other/Unknown	12 (28.6%)
**BMI**	25.4 (22.0–30.2)
**Obesity (BMI ≥30)**	11 (26.2%)
**Cardiovascular Risk Factors/ Cardiovascular Disease**	**No. of patients (%)**
Hypertension	26 (61.9%)
Diabetes mellitus	13 (31.0%)
Cerebrovascular accident	8 (19.0%)
Chronic heart failure	3 (7.1%)
Myocardial infarction	7 (16.7%)
Peripheral vascular disease	7 (16.7%)
**Pulmonary Comorbidities**	**No. of patients (%)**
Asthma	6 (14.3%)
COPD	4 (9.5%)
OSA	2 (4.8%)
Any pulmonary comorbidity	10 (23.8%)
**Smoking (current/ former)**	13 (31.0%)
**Transplant**	5 (11.9%)
**Charlson Comorbidity Index**	4 (2–5)
**Nursing Home Residents**	5 (11.9%)

**BMI:** Body Mass Index, **IQR:** Interquartile range, **No:** number.

The patients had a median duration of symptoms of 6 days (IQR 2–10) prior to the initial positive testing for SARS-CoV-2. During the 1 month follow-up, 10 of 42 included patients (23.8%) required intubation, of whom 3 (30.0%) remained hospitalized with a tracheostomy to ventilator and 4 (40.0%) were extubated and discharged from the hospital. Three patients died after intubation and another 3 were discharged to hospice without ever being intubated (mortality rate 14.3%). The remaining patients had all been discharged.

The included patients had between 1 and 10 repeat positive tests. The median number of repeat tests for each patient was 2 (IQR 2–3), with 18 (42.9%) patients having at least 3 positive tests during the follow-up period. Initial repeat tests were performed at a median of 7 days after the first test (IQR 4–12). Twenty-six patients (61.9%) had repeat testing in anticipation of discharge from the hospital for disposition planning, and 11 patients (26.2%) had repeat testing because a positive SARS-CoV-2 test within 72-h was a requirement for inclusion in a clinical trial. Four patients (9.5%) were discharged and had a repeat positive test for SARS-CoV-2 when they presented again to the emergency department. One patient with acute lymphoblastic leukemia had repeat testing at the time of discharge in anticipation of receipt of chemotherapy. The median initial SOFA score of patients was 2 (IQR 2–3). SOFA Score at the time of repeat testing decreased by at least 2 points compared to the initial testing in 15 patients (35.7%), and increased by at least 2 points in 4 patients (9.5%). The rest had a stable SOFA score between the 2 testings or a difference by one point.

**Fig 2:**
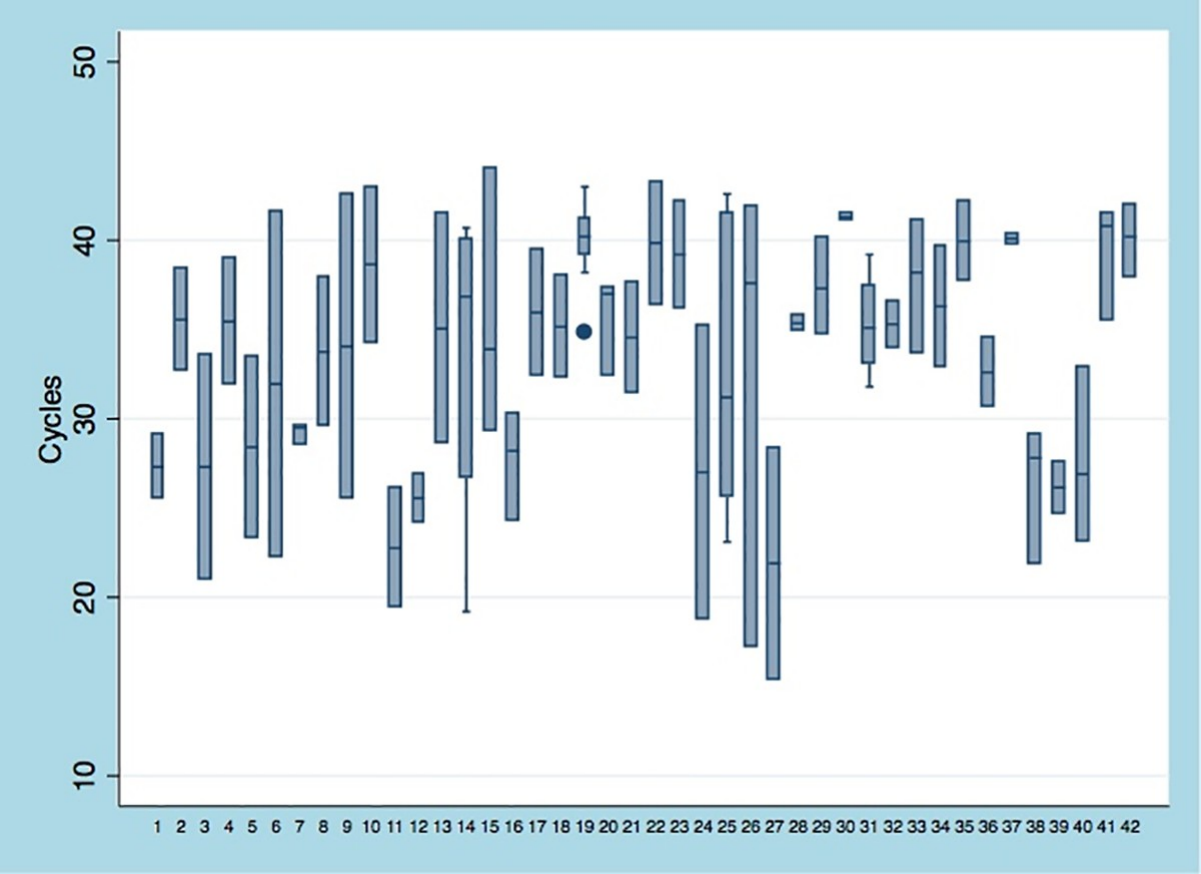
A graph of the Cycle threshold (Ct) values of the of Cepheid Xpert^®^ Xpress SARS-CoV-2 assay measured on repeat screening of the 42 included patients.

**Fig 3:**
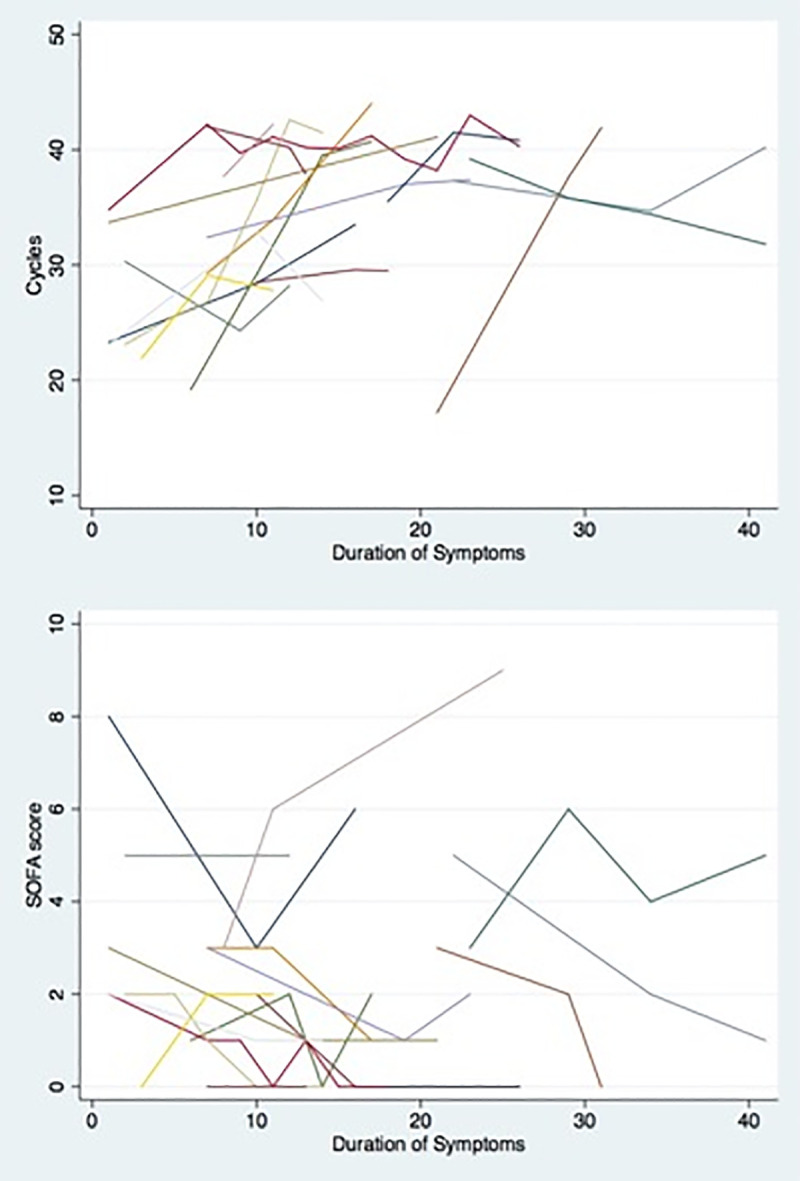
Trend of A. The respective trend of cycles threshold (Ct) of Cepheid Xpert1 Xpress SARS-CoV-2 assay and B. Sequential organ failure assessment (SOFA) scores among patients with at least 2 repeat screenings and a minimum of 2 point difference on SOFA score.

The median initial Ct value among all included patients was 30.5 (IQR 23.5–34.9), while the first repeat value was 36.8 (IQR 33.1–39.8). A graph of the Ct values measured among the 42 included patients is shown in Fig 2. A change in Ct value of 3.0 or more was observed in 31 patients during the first repeat PCR test. For 26 patients (61.9%), the Ct value increased by 3.0 cycles, and for 5 patients (11.9%) decreased by the same margin. Of the 18 patients who had at least 3 screenings, 16 exhibited an either gradually increasing or decreasing trend on the Ct values or had fluctuations that were not more than 3.0 cycles from the immediate prior value (Fig 2). In 1 patient, the Ct value initially decreased by a value of 6.0 cycles on the first repeat test before increasing by a value of 3.9 on the second repeat test. This was a lung transplant recipient, receiving azithromycin and hydroxychloroquine during the 7 days between the 1^st^ and the 2^nd^ tests, with his oxygen requirements increasing up until the day prior to the third test (Venturi mask: FiO2 50%), and then gradually decreasing to room air over next 5 days. Another patient had an initial Ct value of 23, which initially increased to 33 before a third value of 27. This was a patient with multiple medical comorbidities, who had completed a course of hydroxychloroquine between 1^st^ and the 2^nd^ tests, and remained hospitalized for waxing and waning mental status until he was discharged home with hospice services soon after the 3^rd^ test was performed. The SOFA scores of these 18 patients is presented in Fig 3.

**Fig 4:**
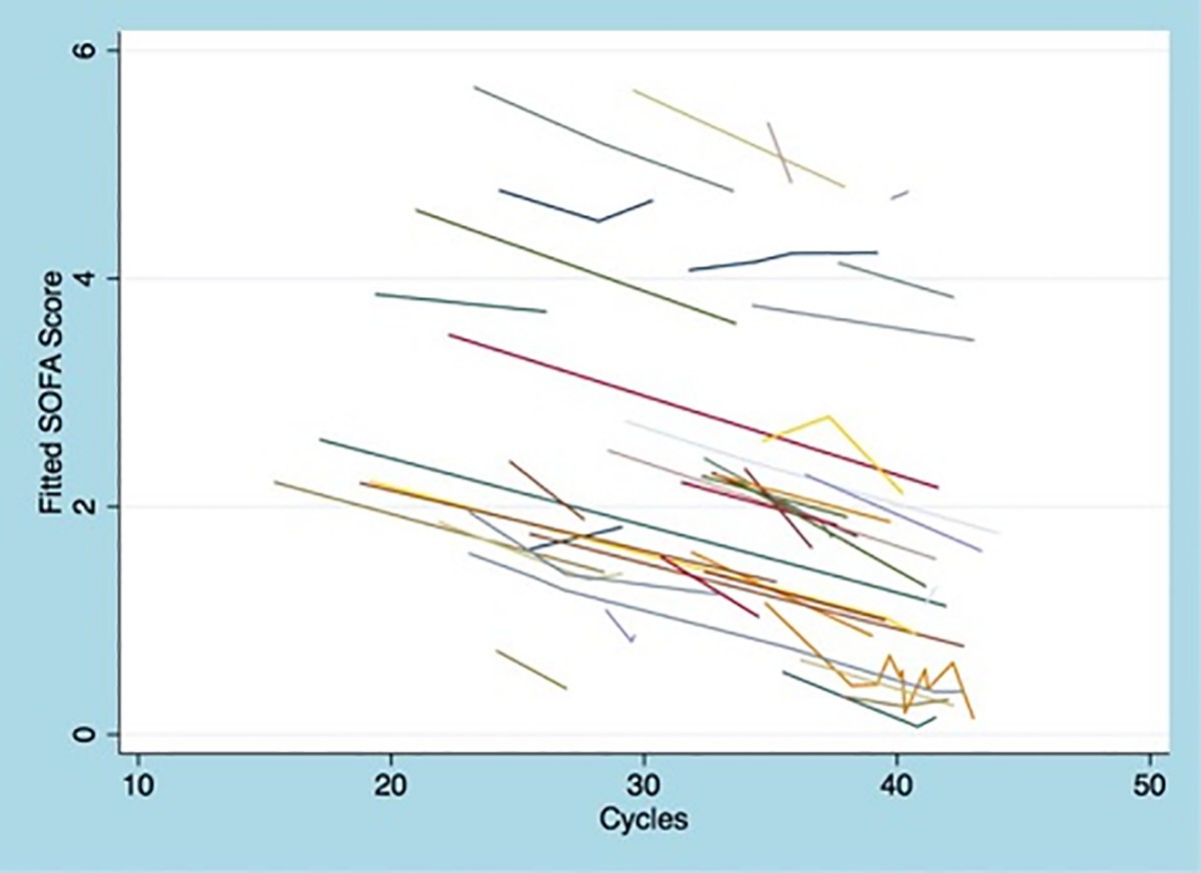
Graph of the fitted SOFA scores based on the cycle threshold values per patient.

The mixed-effects linear regression model showed an inverse correlation between the Ct values and patient’s change in clinical status. An increase in Ct value by 1 correlated associated with a decrease in SOFA score by 0.05 (SE 0.02; p<0.05) after adjusting for the duration of symptoms. Thus, decrease in viral load over time was associated with clinical improvement. A graph of the fitted SOFA scores based on the Ct values per patient is presented in Fig 4.

## Discussion

In this study, we examined the correlation between the SARS-CoV-2 genomic load and the change in clinical status, as reflected by the SOFA score, among patients who had repeat testing with the Cepheid Xpert^®^ Xpress SARS-CoV-2 assay. We identified 42 patients who were diagnosed with COVID-19 pneumonia between March 31^st^, 2020 to April 10^th^ and had repeatedly tested positive over a month follow up period between 2–11 times. We found a negative correlation between the patients’ clinical status and change in the genomic load after adjusting for the duration of symptoms. An increase in Ct value by 3, which represents an approximate order of magnitude difference in genomic load, correlated with a 0.15 improvement on SOFA.

Case series of patients with COVID-19 pneumonia who were followed over a long observation period have suggested a prolonged viral shedding up to 6 weeks for patients with mild to moderate disease, and a median viral shedding of 31 days from illness onset among patients with severe infection [12, 13]. However, the usefulness of the repeatedly positive results in clinical decision making in the individual patient level remains unclear. Attention has been recently drawn on the importance of the genomic load of the virus as reflected by the Ct values of the RT-PCR assays in interpreting the test results [14], and the Infectious Diseases Society of America has emphasized the need for a quantitative test for SARS-CoV-2 to be used as a prognostic marker [6]. Our findings validate the above and suggest that an increasing Ct value in sequential tests may be of prognostic value for patients diagnosed with COVID-19 pneumonia.

As this study was a retrospective cohort analysis, alternative explanations for the observed correlation may exist. The decision about which patients were tested more than once during the follow-up period, as well as the intervals between sequential tests, were made to facilitate individual-level clinical management. A majority of patients were tested prior to disposition to facilitate discharge planning [15]. It can be reasonably assumed that patients tested prior to discharge would have an overall improved clinical status as compared to when they were admitted. Recent studies have suggested that increasing Ct values might reflect the natural course of viral replication in the nasopharynx, which was found to decline after the first 5 days of detection [16]. However, the observed inverse correlation between the patients’ clinical status and change in the genomic load remained after adjusting for the duration of symptoms. As such, even though a causal relationship between Ct values and clinical status cannot be derived from this study, our data suggest that such an association might exist. Also, the 14.3% mortality rate observed among our cohort is remarkably similar to the 14.6% reported recently in a cohort of 4,103 patients diagnosed with COVID-19 at NYU Langone center [17], supporting that our cohort was representative of the patient population with COVID-19 in our institution. Prospective studies with a pre-specified interval for repeat testing are required to elucidate this further.

Upper respiratory specimens are used as the test of choice to diagnose COVID-19 and to detect shedding of the virus [6]. The minimally invasive nature of the test and its availability among all patients diagnosed with COVID-19 pneumonia provide actionable results for prioritization of treatment and inclusion in clinical trials. It is reasonable to assume that Ct values obtained from lower respiratory samples may achieve a higher level of correlation with clinical status, as viral replication theoretically can persist longer or cease earlier in the lower respiratory tract [18]. However, currently, there are no FDA approved assays for detection of SARS-CoV-2 from lower respiratory samples, and it is unlikely that such samples will be regularly obtained in the routine clinical care of patients with COVID-19, limiting their value pragmatically. However, the way SARS-CoV-2 viral load correlates with culturable virus needs to be determined.

Limitations of the current study are acknowledged and arise from its retrospective observational nature. Inter-operator variability in specimen collection techniques could have resulted in variation in the amount of specimen collected from each patient. This would be expected to add random error in the analysis and would have favored a lack of association. Additionally, we believe it is unlikely that such a variation results in a significant alteration of genomic load here because in 88.9% of patients with 3 or more tests, we observed Ct results either within an order of magnitude or a stable increase or decline in sequential tests. Only the 2 patients described in the Results exhibited a difference of more than 3.0 cycles in Ct values that initially decreased and then increased. Although, it would be important to elucidate the factors that led to that change, the limited number of patients did not allow for valuable conclusions. Last, this study relies on Ct values obtained through a single assay to ensure comparability between tests in terms of targets, amplification efficiency and dynamic range. Though it seems reasonable to speculate that similar associations between Ct and clinical trends might apply to other assays in a broad sense, we advise caution with generalizing the outcomes across different RT-PCR methods.

In summary, we showed that among a population of patients diagnosed with COVID-19 pneumonia, improvement in clinical status was associated with a decreasing SARS-CoV-2 genomic load as reflected by increasing Ct values in sequential RT-PCR nasopharyngeal swabs. Our results point to the potential predictive ability of a repeat SARS-CoV-2 genomic load in disease outcome. We stress the importance of future studies with repeat testing at predetermined intervals, validating results across different RT-PCR platforms, and determining factors for variations in genomic load in the individual patient to further examine this association before Ct values of repeat testing can be recommended in routine clinical care to monitor clinical outcomes.

## Supporting information

S1 Raw data (XLSX)
